# Identification of Tumor Antigens and Immune Subtypes in Lung Adenocarcinoma for mRNA Vaccine Development

**DOI:** 10.3389/fcell.2022.815596

**Published:** 2022-02-21

**Authors:** Ran Xu, Tong Lu, JiaYing Zhao, Jun Wang, Bo Peng, LinYou Zhang

**Affiliations:** ^1^ Department of Thoracic Surgery, The Second Affiliated Hospital of Harbin Medical University, Harbin, China; ^2^ Harbin Medical University, Harbin, China

**Keywords:** lung adenocarcinoma, mRNA vaccine, tumor antigens, antigen presentation, individualized treatment

## Abstract

Cancer vaccines are emerging as a viable strategy for cancer treatment. In the current study, we screened for genes associated with the prognosis of patients with lung adenocarcinoma and positively correlated with antigen-presenting cell infiltration and identified KLRG1 and CBFA2T3 as potential tumor antigens for mRNA vaccines in lung adenocarcinoma (LUAD). Further analyses of immune subtypes revealed that patients with early-stage LUAD, high immune cell infiltration, high immune checkpoint expression, and low tumor mutation burden might benefit from mRNA vaccination. Moreover, we identified four biomarkers that can be used to assess mRNA vaccination suitability. We also identified potentially sensitive anti-cancer drugs for populations not suitable for vaccination by means of anti-cancer drug susceptibility prediction. Overall, we provided a new perspective for mRNA vaccine treatment strategies for LUAD and emphasized the importance of precise and personalized treatments.

## Introduction

Lung cancer is the leading malignant disease death cause (Siegel et al., 2021) with a 5-years survival rate of 4–17% (Hirsch et al., 2017). Lung adenocarcinoma (LUAD) is the most common lung cancer pathological subtype and great progress has been made recently regarding individualized and precise treatments. To date, surgery is the primary treatment for early-stage LUAD but surgical opportunities are often missed in asymptomatic patients (Tanoue et al., 2015; Abbas 2018). Therefore, alternatives are required for those lung cancer patients that cannot undergo surgery. In these cases, other important treatment strategies can be used, such as targeted therapy (e.g., targeting EGFR mutations) and immunotherapy (Mayekar and Bivona 2017; Osmani et al., 2018). However, resistance to EGFR-TKI or low immune checkpoint expression remains challenging (Wu and Shih 2018; Santarpia et al., 2020). Hence, a new strategy for the precise treatment of LUAD needs to be developed.

Recently, the development of vaccines to treat cancer has received significant attention from researchers worldwide (Sullenger and Nair 2016). The main challenge in cancer vaccine development lies in finding specific and personalized tumor cell antigens that will be activated and prepare a patient’s immune system to recognize cancer cells, eliminating immune escape possibilities (Bowen et al., 2018; Sahin and Tureci 2018). Among the different types of vaccines, mRNA vaccines have advantages such as being easy to prepare; mRNA does not integrate into the host’s genome and they can be degraded by RNases in cells, increasing their long-term safety (Pardi et al., 2018). The clinical safety of mRNA vaccines was indeed confirmed after their vast clinical application in COVID-19 prevention (Polack et al., 2020). Currently, several mRNA cancer vaccines are undergoing clinical trials (Li et al., 2014; Wang et al., 2018; Cafri et al., 2020), but no relevant reports on mRNA vaccines for LUAD have been reported.

In the current study, we investigated potential tumor antigens of LUAD that could be used to develop mRNA vaccines. We found two potential tumor antigens involved in antigen-presenting cell infiltration in LUAD and associated with a better prognosis. Moreover, we determined the characteristics of patients eligible for mRNA vaccination after grouping populations according to immune subtypes. Altogether, our findings provided new ideas for mRNA vaccines development and personalized treatment strategies for LUAD.

## Methods

### Identification of Significantly Altered Copy Number Variant Chromosomal Regions, Gene Mutations, and Prognosis-Related Genes

To analyze the copy number variation of candidate genes in TCGA-LUAD, we used TCGAbiolinks R package (Colaprico et al., 2016) to download “Masked Copy Number Segment” data, and the GISTIC 2.0 software (https://cloud.genepattern.org/) (Mermel et al., 2011)was used to identify chromosomal regions with significant copy number variations. The threshold for significant amplifications and/or deletions was set as a q-value < 0.01. The R package “maftools” (Mayakonda et al., 2018) was used to visualize the mutation landscape. To analyze the functional enrichment of genes affected by copy numbers variations, the “clusterprofiler” R package (Yu et al., 2012) was used. Analyses of all prognosis-related genes in the TCGA-LUAD cohort were performed using the GEPIA2 database (http://gepia2.cancer-pku.cn/#survival). Patients was grouped according to the median value of each gene expression, and *p-value* < 0.05 was considered significant for prognosis.

### Identification of Potential mRNA Vaccine Antigens in Lung Adenocarcinoma

Correlation analyses between the gene expression of potential vaccine antigens and immune cells were conducted using the TIMER database (https://cistrome.shinyapps.io/timer/). The GEPIA2 database (Tang et al., 2019) was used to analyze antigen gene expression and survival in LUAD patients. The expression of antigen genes in patients in different stages was visualized using the “ggplot2” R package (https://CRAN.R-project.org/package=ggplot2).

### Identification of Immunophenotyping in Lung Adenocarcinoma Patients

After retrieving immune-related gene sets from the IMMPORT database (https://www.immport.org/shared/home), immune-related gene expressions were extracted from the TCGA-LUAD cohort FPKM data. The “ConsensusClusterPlus” R package (Wilkerson and Hayes 2010) was used to distinguish the immune subtypes. The clustering algorithm used the k-means of Euclidean distance, the total number of subsampling is set to 50, 80% of the total sample proportion is selected for each resampling, and the maximum number of clusters is set to 9. The optimal K was determined using the elbow method and ensuring that the number of patients in each cluster was ≥100. Survival analysis between clusters was performed using the “survival” R package. Hallmark gene set analysis for each sample was conducted using the “GSVA” R package (Hanzelmann et al., 2013), and the Hallmark gene set was from the Msigdb database (Liberzon et al., 2015). Poisson distribution was set up during the analysis and the pathway contained at least 10 genes. Heat maps were plotted using the “pheatmap” R package (https://CRAN.R-project.org/package=pheatmap).

### Analysis of Tumor Immune Infiltration Microenvironment

The “Estimate” algorithm was used to calculate the immune stromal cell score for each sample. Since the Estimate package does not output tumor purity by default for data from the sequencing platform, the tumor purity of the samples was calculated manually according to the previously published literature (Yoshihara et al., 2013).

Tumour purity=cos (0.6049872018+0.0001467884* ESTIMATE score)

The gene sets used to assess infiltrating immune cells for all samples were obtained from previously published articles (Supplementary Table S1) (Charoentong et al., 2017) Immune checkpoints and immunogenic cell death modulators genes were referred to previously published articles (Huang et al., 2021a).

### Identification of Mutations Among Clusters

The “maftool” R package was used to visualize the gene mutation landscape among clusters and to calculate the tumor mutation burden for each sample. Additionally, the MutsigCV software was used to identify LUAD driver mutations in the cohort (Lawrence et al., 2013). Human Genome Assembly GRCh38 was used as the reference genome in the analysis (https://www.ncbi.nlm.nih.gov/grc/human/data?asm=GRCh38). Significant mutations were defined as a *q*-value < 0.05, and driver mutated genes were chromosomally mapped using the “RCircos” R package (Zhang et al., 2013).

### Weighted Gene Co-expression Network Analysis

The WGCNA was performed using the “WGCNA” R package (Langfelder and Horvath 2008) and normalized expressions of immune-related genes. After clustering the samples, no outlying samples were found. Hence, all samples were included in subsequent analyses. First, the Person correlation coefficient between any two genes is calculated and a similarity matrix is built by the results. The optimal soft threshold power “*β* = 3” is then obtained by unitary linear regression matching so that R2 > 0.85, and the similarity matrix is transformed into an adjacency matrix according to the optimal soft threshold power, which is then transformed into a topological overlap matrix (TOM). Eventually, we used 1-Tom as the distance to cluster the genes. The minimum number of genes per module was set to 20. To further analyze the modules, we calculated the dissimilarity of the module eigengenes, set the sensitivity to 2, and no modules were merged. Nine modules were identified after the soft threshold was set at three. Then, the correlation between modules and cluster classification were analyzed. KEGG functional enrichment analysis of genes in modules of interest was performed using “Clusterprofiler”. The predictive ability of each gene for prognosis was assessed using ROC curves plotted by the “timeROC” R package (https://CRAN.R-project.org/package=timeROC). Genes with an area under the curve (AUC) > 0.6 were considered as better predictive biomarkers.

### Anticancer Drug Sensitivity Analyses

The Genomics of Drug Sensitivity in Cancer (GDSC) database (Yang et al., 2013) is a database for therapeutic biomarker discovery in cancer cells. The R package “pRRophetic” (Geeleher et al., 2014)was used to construct a ridge regression model according to the GDSC cell line expression and TCGA-LUAD expression profiles, to assess the sensitivity of each sample to multiple anti-cancer drugs. Immunotherapy response was predicted using the TIDE (Tumor Immune Dysfunction and Exclusion) online tool (http://tide.dfci.harvard.edu/) (Jiang et al., 2018; Fu et al., 2020).

## Results

### Identification of Potential Tumor Antigens of Lung Adenocarcinoma

**FIGURE 1:**
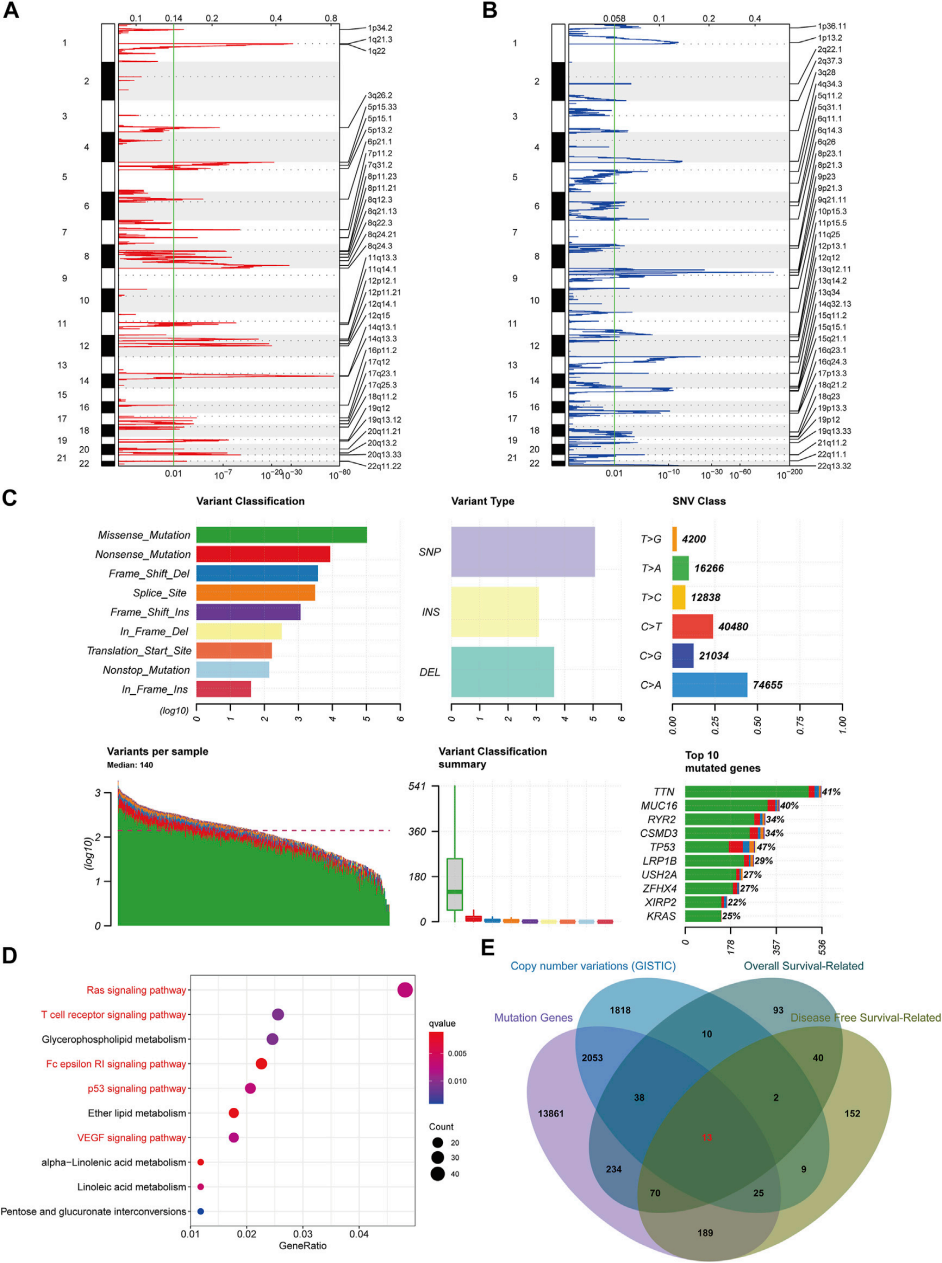
Screening for potential tumor antigens in lung adenocarcinoma. **(A)** Identification of chromosomal copy number amplification regions. **(B)** Identification of chromosomal copy number deletion regions. **(C)** Summary of gene mutations in lung adenocarcinoma. **(D)** KEGG functional enrichment analysis of genes in regions of chromosomal copy number variation. **(E)** Identification of instability genes associated with prognosis.

Variations in copy number and mutations caused by genomic instability are crucial in cancer development (Bailey et al., 2021). Also, tumor cell heterogeneity caused by these variations drives phenotypic adaptation during tumor progression, leading to drug resistance (Sansregret et al., 2018). Therefore, to find tumor-specific antigens in LUAD as potential mRNA vaccine targets, we first investigated the chromosomal variation landscape in LUAD using the GISTIC software (Mermel et al., 2011). We considered genes affected by regions with significant chromosomal amplification and/or deletion as potential mRNA vaccine targets (Figures 1A,B). Moreover, we analyzed the landscape of gene mutations in LUAD patients. We found that missense and nonsense mutations occurred at higher frequencies and a single nucleotide variant was the most frequent mutation type (Figure 1C). Next, we performed a KEGG enrichment analysis of the copy number variant genes. Results showed that these genes were involved in the regulation of different cancer and immune-related pathways, demonstrating that these genes could be used as potential targets for cancer mRNA vaccines (Figure 1D). We also used all genes with mutations in the TCGA database as candidate targets for mRNA vaccines. Relevant genes retrieved from the GEPIA database that could affect patients’ overall survival and disease-free survival were used for screening. Fianlly, we obtained 13 LUAD-specific antigens (TTK, BUB1B, ASB2, HJURP, CBFA2T3, PTGFRN, HSPA4, ST6GAL1, CENPU, KLRG1, GTF3C6, OIP5, VDAC3) that could affect patients prognosis (Figure 1E). These tumor antigens hold promise as new therapeutic targets against immune escape of tumor cells.

### Potential Antigen Expression Correlates With Patient’s Survival and Antigen-Presenting Cells

**FIGURE 2:**
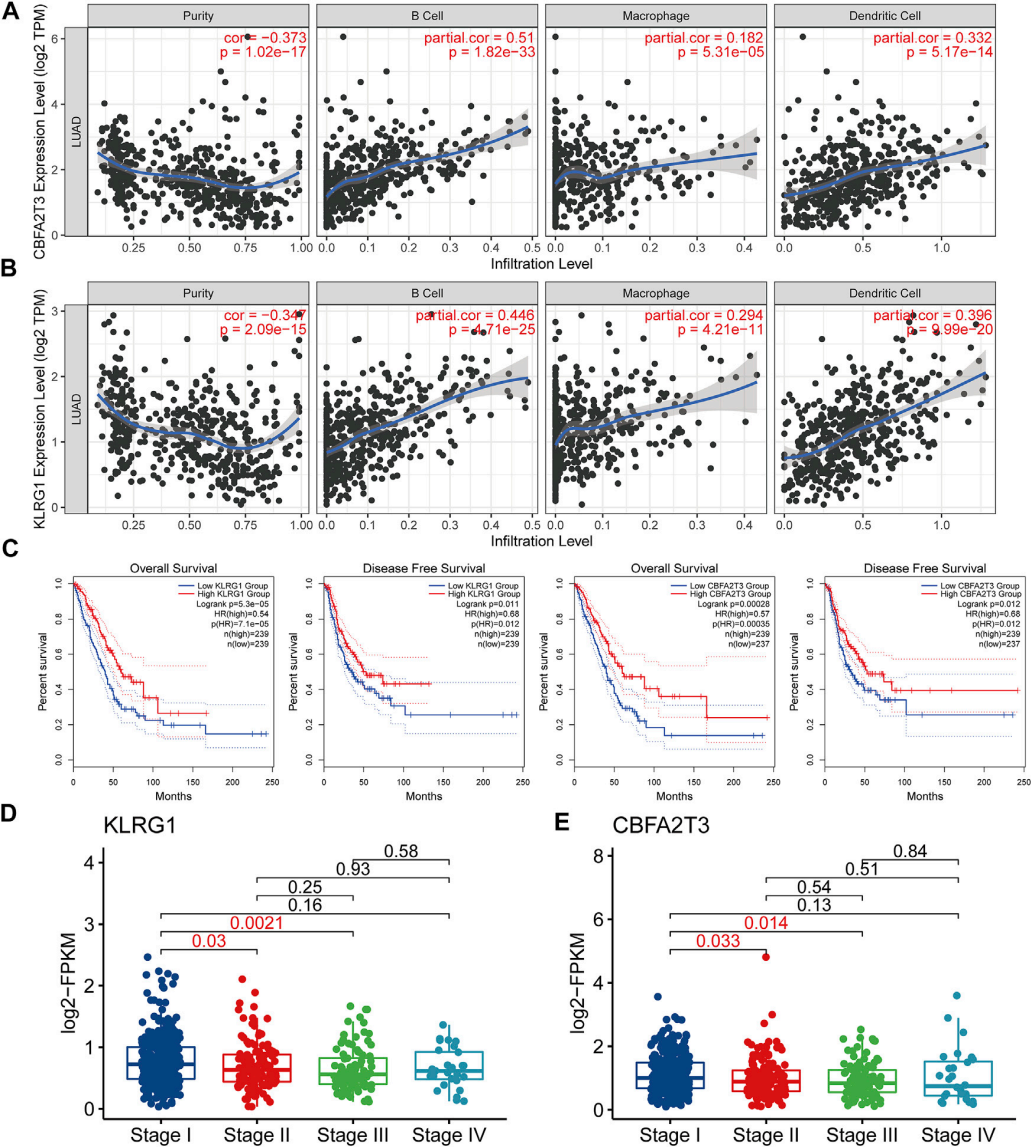
Identification of tumor antigens in lung adenocarcinoma that can be used to develop mRNA vaccines **(A)** The expression of CBFA2T3 is positively correlated with the infiltration of a variety of antigen-presenting cells. **(B)** The expression of KLRG1 is positively correlated with the infiltration of a variety of antigen-presenting cells. **(C)** CBFA2T3 and KLRG1 are associated with overall survival and disease-free survival in patients with lung adenocarcinoma. **(D–E)** Expression of CBFA2T3 and KLRG1 in different stages of patients with lung adenocarcinoma.

**FIGURE 3:**
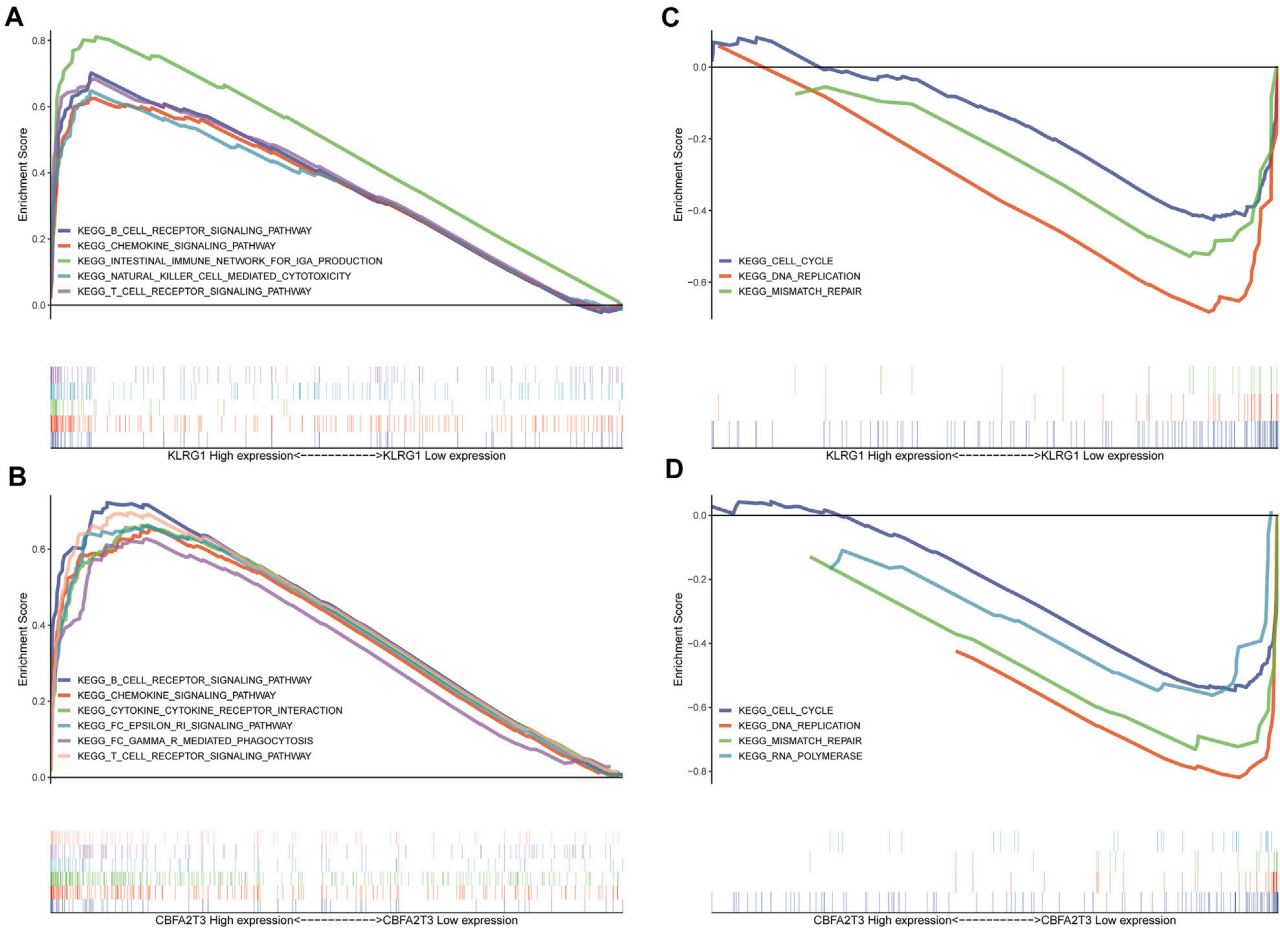
Expression of tumor antigens is associated with a variety of immune pathways and cell cycle pathways **(A–B)** Patients with high expression of KLRG1 and CBFA2T3 have more active immune-related pathways. **(C–D)** Patients with low expression of KLRG1 and CBFA2T3 have a more active cell cycle.

The immunogenicity of mRNA vaccine is the key to whether it can effectively activate the immune system. The higher immunogenicity represents that it can be recognized and processed by antigen-presenting cells and presented to T cells, which plays an important role in the immune recognition, immune response, and immune regulation. Antigen presenting cells are a major route for mRNA vaccine-mediated lymphocyte generation of immune memory and anti-tumor immunity (Cafri et al., 2020; Xu et al., 2020). Therefore, we investigated the relationship between the antigen candidates obtained above and three immune cell types that act as antigen-presenting (B cells, dendritic cells, and macrophages) to screen for optimal LAUD antigens. Dendritic cells play a vital role in the initiation and regulation of innate and adaptive immunities (Wculek et al., 2020). Moreover, B cells act as antigen-presenting cells by mediating memory T cells activation (Popi et al., 2016). We found that the expression of two, CBFA2T3 and KLRG1, of the 13 antigen candidates was significantly positively correlated with APCs expressions (Figures 2A,B). This result suggested that mRNA vaccines based on these two antigens might induce immune system activation after injection. After grouping based on the expression of these two potential antigens in LUAD patients, we observed that patients with high CBFA2T3 and KLRG1 expressions had a better prognosis. This also indicated that patients with high expression of these two genes may have a more conducive immune microenvironment for survival (Figure 2C). Then, we investigated the expression of KLRG1 and CBFA2T3 in different tumor stages (Figures 2D,E). The results show that these two genes are highly expressed in early-stage patients, reflecting that mRNA vaccines designed based on these two tumor antigens may produce better immunogenicity in early-stage patients. After grouping the cohorts according to the expression of these two potential antigens and performing a GSEA-based KEGG enrichment analysis, we observed an enrichment of multiple immune-related pathways in the highly expressed population (Figures 3A,B). In contrast, their low expression was associated with active cell cycle pathways (Figures 3C,D). Compared with other disease vaccines, therapeutic cancer vaccines mainly play a therapeutic role rather than prevention (DeMaria and Bilusic 2019), which should be clarified for the appropriate population, so we next identified the characteristics of lung adenocarcinoma patients suitable for mRNA vaccination.

### Identification of Immune Subtypes in Lung Adenocarcinoma Patients

**FIGURE 4:**
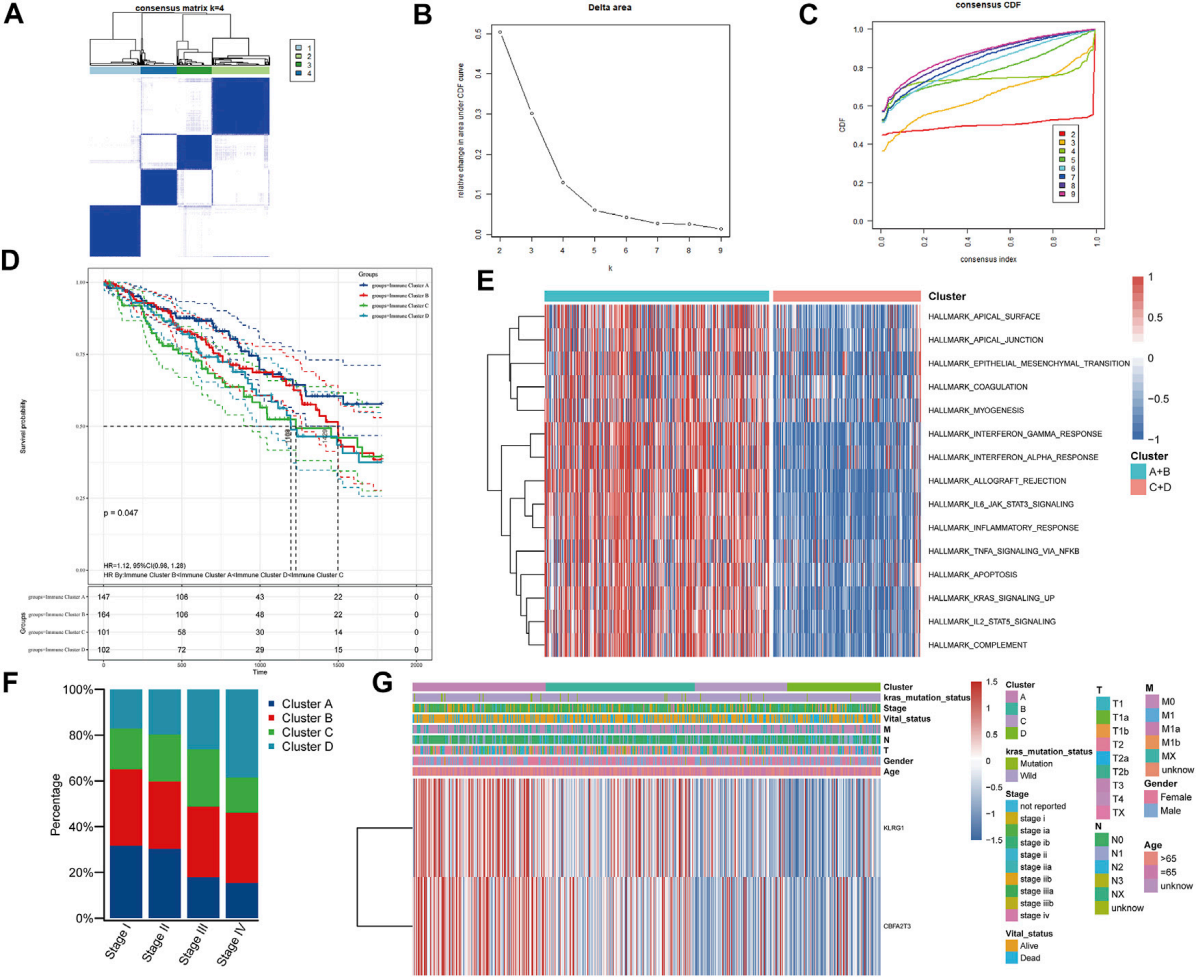
Identification of immune clusters in patients with lung adenocarcinoma. **(A–C)** Patients with lung adenocarcinoma were divided into four immune clusters according to the expression of immune-related genes. **(D)** Survival analysis of patients with different immune clusters. **(E)** GSVA enrichment analysis of cancer Hallmarks in patients with different immune clusters. **(F)** Distribution of immune clusters in patients with different stages. **(G)** Heat map of tumor antigen expression and clinical parameters.

Immune subtypes can reflect the current tumor microenvironment status of patients and the patient’s current immune status correlates with the effect of immune-related therapy (Ye et al., 2021a). Hence, we used immune-related genes in the TCGA-LUAD cohort to identify different patient populations and to assess their suitability for mRNA vaccination. Using consensus clustering, we identified four LUAD patients subtypes with different immune characteristics (Figures 4A–C). Clusters A and B had the best prognosis among the four subtypes, suggesting that the LUAD tumor microenvironment might affect prognoses (Figure 4D). The enrichment analysis of cancer hallmarks comparing Clusters A and B (better prognoses) to C and D subtypes (worse prognoses) also showed multiple immune-related pathways enriched for A and B (Figure 4E). Therefore, vaccination of Clusters A and B patients might produce a robust immune response. Additionally, after analyzing patients in different stages, we observed a higher proportion of Clusters A and B in patients with stages I and II, consistently with the results in Figures 2D,E. These results demonstrated that early-stage tumors patients are more sensitive to vaccination (Figure 4F). Notably, KLRG1 and CBFA2T3 were highly expressed in Clusters A and B (Figure 4G). Our results indicate that patients with Cluster A and B have an active current immune status and higher expression of the two tumor antigens identified. Cluster A and B may therefore have better immunogenicity for mRNA vaccines. Altogether, these results suggested that patients from Clusters A and B are more suitable for mRNA vaccination using these antigens.

### Characteristics of the Tumor Immune Microenvironment in Various Immune Clusters

**FIGURE 5:**
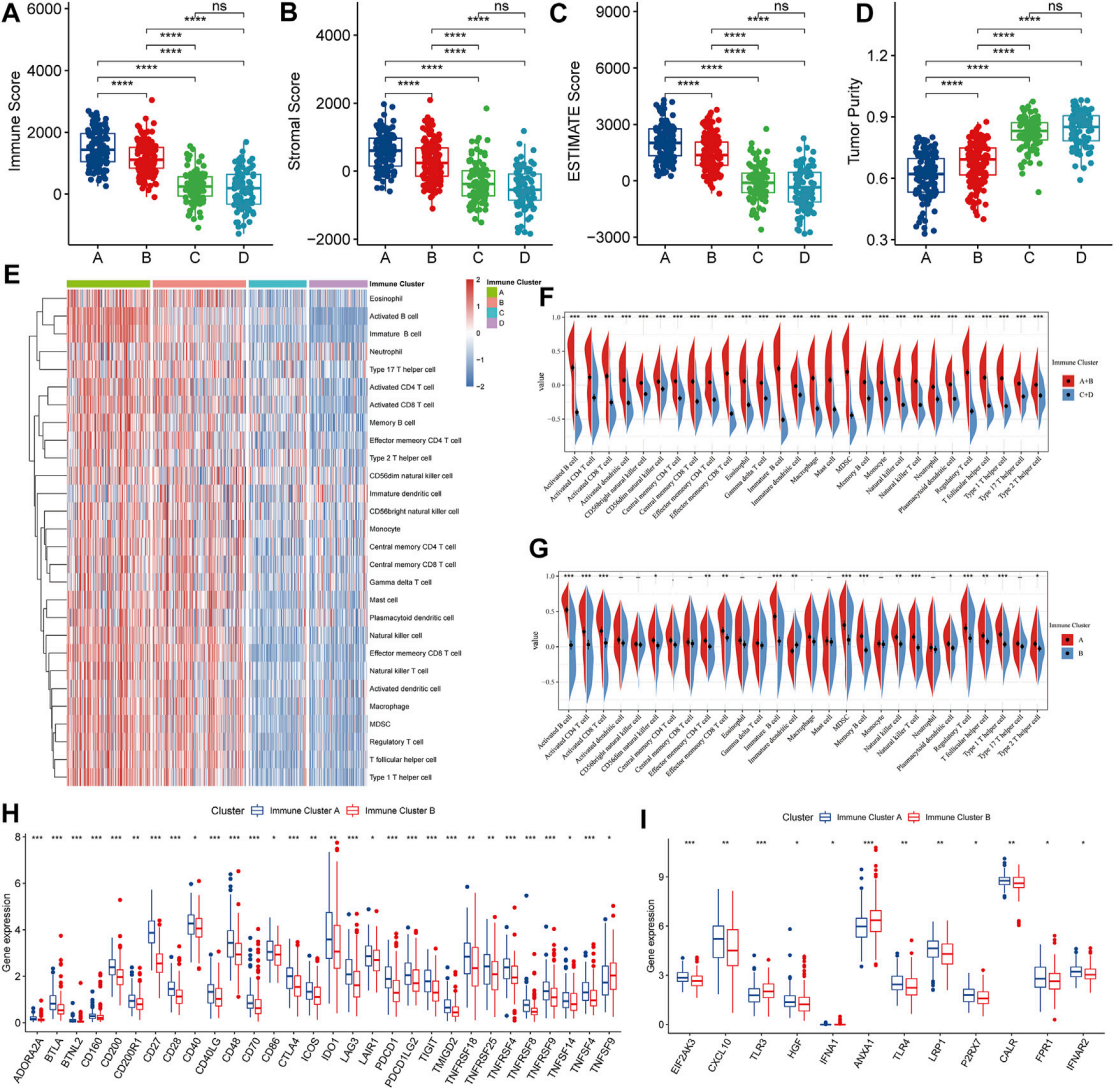
Identification of tumor immune cell infiltration in patients with different clusters. **(A–D)** Immune-stromal scores and tumor purity of patients in each cluster were assessed by the ESTIMATE algorithm. **(E)** Heat map of immune cell infiltration levels assessed based on ssGSEA enrichment. **(F–G)** Comparison of immune cell infiltration levels between clusters. **(H)** Comparison of immune checkpoint expression between clusters. **(I)** Immunogenic cell death modulators genes expression comparison between clusters.

To further characterize the tumor microenvironment of each immune cluster, we first assessed immune and stromal scores using the ESTIMATE algorithm (Figures 5A–D). Clusters A and B had higher immune and stromal scores, while tumor purity was lower, suggesting that the proportion of tumor-infiltrating immune cells was higher in these samples. Also, we quantified various infiltrating immune cells using previously published immune cell gene sets (Figure 5E). After contrasting Clusters A and B with C and D, the data suggested that A and B might behave as “hot” immune subtypes, while C and D as “cold” subtypes (Figure 5F). Additionally, along with high KLRG1 and CBFA2T3 expressions (Figure 3G), clusters A and B may have a higher responsiveness to mRNA vaccination. Further, we contrasted Clusters A and B and showed that most immune cells were more infiltrated in Cluster A than in B. Therefore, Cluster A patients might be the most appropriate population to be vaccinated (Figure 5G). Moreover, we found a range of immune checkpoints and high expression of immunogenic cell death modulators in Cluster A, indicating that this population would also have better effects on immunotherapy (Figures 5H,I).

### Relationship Between Immune Clusters and Tumor Mutation Burden

**FIGURE 6:**
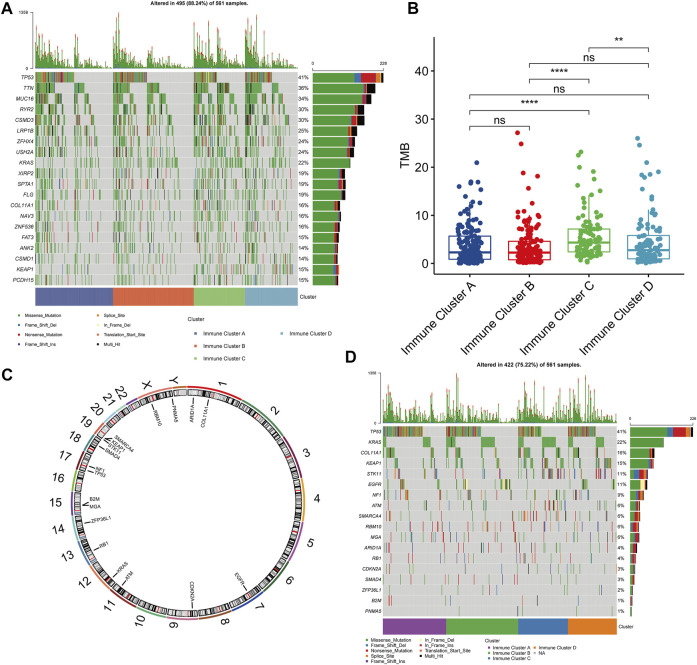
Gene Mutation Landscapes in Patients with Different Clusters. **(A)** Top 20 genes in mutation frequency in all clusters. **(B)** Comparison of tumor mutation burden in different clusters. **(C)** Chromosomal localization of tumor driver mutated genes identified by MutSigCV algorithm. **(D)** Distribution of tumor driver mutant genes in clusters.

Then, we summarized the mutation landscape of all clusters and observed that KRAS had a higher mutation frequency in all of them (Figure 6A). Various studies have reported that KRAS mutations can lead to resistance to EGFR-TKIs that are widely used (Lehmann et al., 2019; McFall et al., 2019; Scheffler et al., 2019). Thus, mRNA vaccines may constitute an alternative treatment strategy for these populations. After calculating the TMB, Cluster A presented lower TMB (Figure 6B). Therefore, this low TMB could be correlated with vaccination suitability. We also used the MutSigCV algorithm to assess tumor-driver gene mutations and determined their location on the chromosome (Figure 6C). After visualizing the driver mutation genes in each patient, we observed that the co-mutation frequency of EGFR and KRAS was rare in Cluster A, indicating that EGFR-TKIs are still a promising treatment strategy for these patients. Hence, we believe that patients should be evaluated, and, for example, target gene sequencing should be performed before considering vaccination (Figure 6D).

### Identification of Immune Gene Co-expression Module in Lung Adenocarcinoma

**FIGURE 7:**
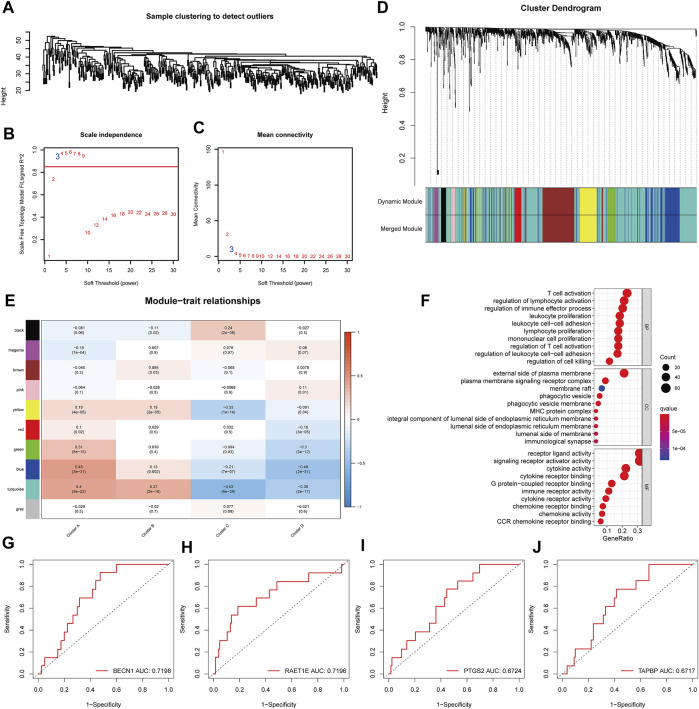
Identification of gene co-expression network and identification of biomarkers in the suitable vaccinated population. **(A–D)** Identification of WGCNA gene modules. **(E)** Correlation analysis between gene modules and immune clusters. **(F)** Gene Ontology (GO) functional enrichment analysis of genes in the blue module. **(G–J)** Four potential prognostic biomarkers in cluster A patients eligible for mRNA vaccination.

Immune-related genes were clustered using the WGCNA algorithm, and “three” was chosen as the soft threshold power based on the scale-free fit index and average connectivity (Figures 7A–C). Ten modules were identified among all immune-related genes (Figure 7D). After investigating the association between modules and phenotypes, we observed that the “blue” module was characteristic of Cluster A (suitable for vaccination) (Figure 7E). After extracting the genes from the blue module, the Gene Ontology (GO) enrichment analysis results showed that these genes were involved in the activation and proliferation of various immune cells (Figure 7F). Further, we investigated the predictive ability of these genes for the prognosis in the Cluster A population. We identified four genes (BECN1, RAET1E, PTGS2, TAPBP) with a good predictive ability for the prognosis (ROC curves AUC >0.6) (Figures 7G–J). Moreover, BECN1 was experimentally validated in a previous article as an important initiator molecule of autophagy and inhibited the growth of lung adenocarcinoma cells (Han et al., 2018) and it also plays a role in the regulation of the immune system (Cui et al., 2016), while PTGS2 was also confirmed as a prognostic biomarker for lung adenocarcinoma (Castelao et al., 2003). Therefore, these four genes could be used as biomarkers after mRNA vaccination.

### Immune Clusters Are Associated With Anti-Cancer Drug Sensitivity

**FIGURE 8:**
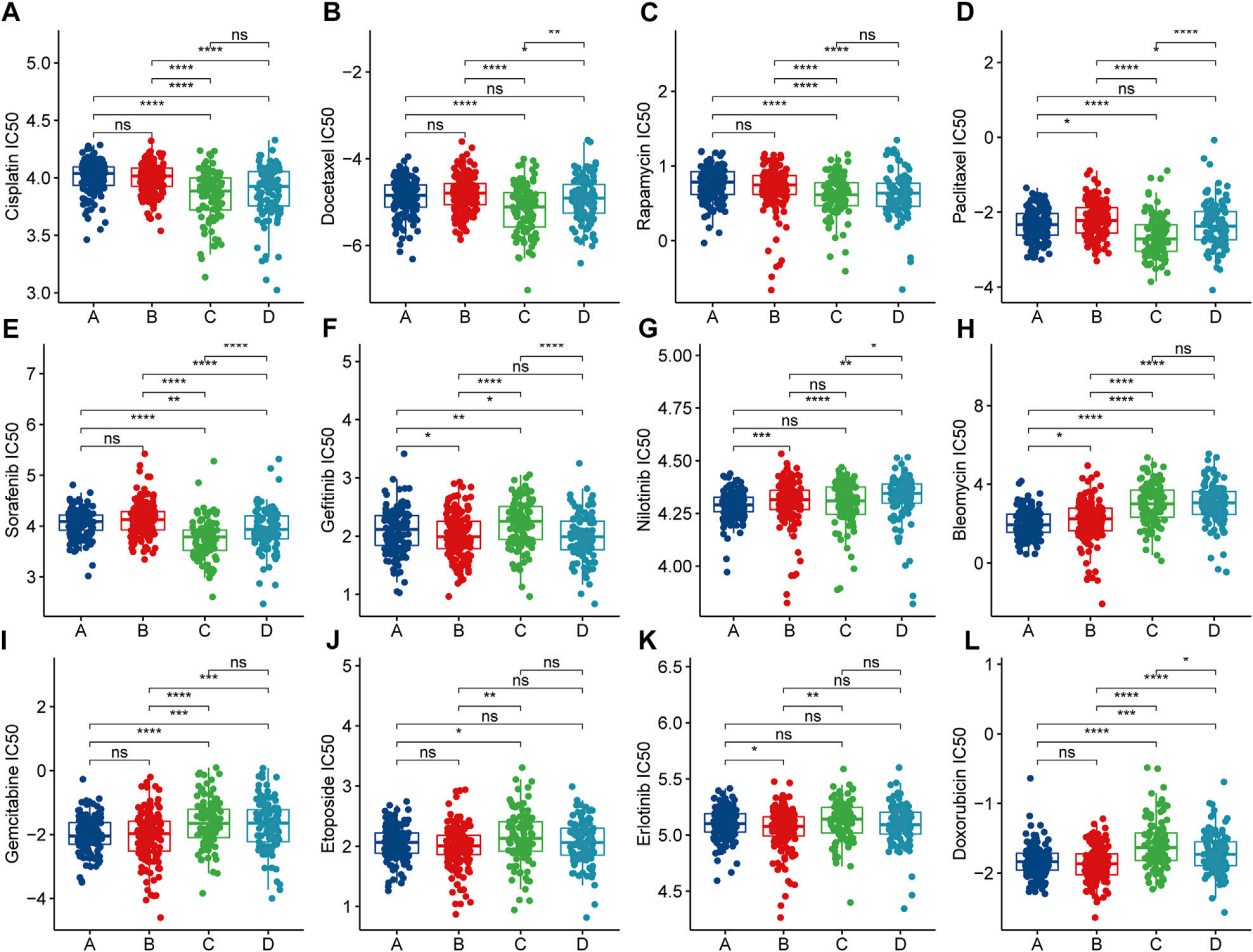
Anticancer Drug Sensitivity Analysis in Patients with Different Clusters. **(A–L)** Half maximal inhibitory concentration (IC50) of multiple anticancer drugs in patients with different clusters.

To explore therapeutic strategies for clusters that are not suitable for mRNA vaccination, we used drug sensitivity data from the Genomics of Drug Sensitivity in Cancer (GDSC) database as a training set to predict common anti-cancer drug sensitivity in TCGA-LUAD cohort samples. Surprisingly, after contrasting clusters, we found that cisplatin - a drug widely used to treat advanced lung cancer - had a lower half-maximal inhibitory concentration (IC50) in clusters not suitable for vaccination, such as C and D (Figure 8A). This implied that Clusters C and D might be chemosensitive. We also found potentially sensitive anti-cancer drugs in Clusters C and D (Figures 8B–E). For Cluster A and B (suitable for vaccination), we observed a low IC50 for gefitinib (Figure 8F). This might be associated with the higher EGFR mutation frequency observed above (Figure 6D). Similarly, we found other potentially sensitive anti-cancer drugs suitable for Clusters A and B (Figure 8G-L). The TIDE score effectively reflects the potential benefit of immunotherapy and is superior to the prediction of immune checkpoint expression and mutations. When we used the TIDE online tool for immunotherapy response prediction in the overall cohort, we found that Cluster A and B tumor immune dysfunction scores were high (Supplementary Figure S1A–B), which reflects that they may not be sensitive to immune checkpoint blockade, so although Cluster A and B have higher immune checkpoint expression, but they may not be effective for immunotherapy. The mRNA vaccine may be a potentially effective alternative therapeutic strategy. Altogether, immune Clusters A and B were not only suitable for mRNA vaccination but also may be sensitive to targeted therapy. On the other hand, immune Clusters C and D might be more sensitive to chemotherapy. Differences in treatment sensitivity between different immune clusters also reflected the heterogeneity among tumors, emphasizing the importance of individualized treatments.

## Discussion

Recently, due to the broad clinical application of mRNA vaccines, their feasibility and safety have been confirmed (Polack et al., 2020). These vaccines have good prospects in cancer treatment, but more research is needed (Sahin and Tureci 2018). Mining tumor antigens with better immunogenicity and immunoreactivity in different cancers is the key to mRNA vaccine development. In the current study, we identified KLRG1 and CBFA2T3 as potential antigenic targets that could be used to develop mRNA vaccines for LUAD. Further analyses of immune subtypes identified a patient population that would benefit the most from vaccination. We also identified four biomarkers (BECN1, RAET1E, PTGS2, and TAPBP) that could be used to monitor vaccine response. Finally, we identified potentially sensitive anti-cancer drugs in the populations unsuitable for vaccination through drug sensitivity analyses. During the study, we found that the patient population with high immune checkpoint expression may not be sensitive to immune checkpoint blockade therapy due to immune system disorders, so mRNA vaccines may be another potential therapeutic strategy, but further studies are needed to verify our view. Overall, we identified two tumor antigens in LUAD - KLRG1 and CBFA2T3 - that could be used to develop mRNA vaccines. Also, we identified potential individualized treatment strategies for patients with different immune subtypes.

Tumor antigens can activate various antigen-presenting cells. Thus, inducing tumor-specific immunity and immune memory can be used in the development of tumor mRNA vaccines (Khan et al., 2021). KLRG1 has been reported as a prognosis biomarker in LUAD patients and is associated with response to immunotherapy (Yang et al., 2021). Additionally, combined blockade of KLRG1 with PD-1 can promote NK and T cell anti-tumor immunity (Tata et al., 2021). On the contrary, there has also been reported that high KLRG1 expression is associated with low proliferation of lung adenocarcinoma cells, however, KLRG1 expression is often low in lung adenocarcinoma cells, and associated with adverse effects of immunotherapy (Yang et al., 2021). The cancer-promoting and cancer-suppressing effects of KLRG1 appear paradoxical in response to immunotherapy and the effects of the cell cycle of the tumor cells themselves. But an article reported that KLRG1+effector CD8+T cells can effectively promote anti-tumor immunity after differentiation and loss of KLRG1 expression (Herndler-Brandstetter et al., 2018). Therefore, we believed that the development of KLRG1 as an mRNA vaccine could help to stimulate KLRG1+Effector CD8+T Cells to differentiate into memory T cells of other lineages and produce effective anti-tumor immunity. Using KLRG1 as an mRNA vaccine can achieve anti-tumor immunity by activating the immune system without altering KLRG1 expression in cancer cells themselves to affect the cell cycle. In our current study, we found a positive correlation between KLRG1 and the infiltration of different antigen-presenting cells, revealing that KLRG1 could induce tumor immunity and immune memory, and could be used as a potential tumor antigen for mRNA vaccines. Also, KLRG1 can be a marker of immune cell senescence, and the expansion of oligoclonal senescent T cells may negatively impact immunotherapy (Ferrara et al., 2021). Therefore, using KLRG1 as a tumor antigen, APCs will be trained to recognize and remove KLRG1-expressing tumor cells and senescent immune cells, becoming a new LUAD treatment strategy. CBFA2T3 has been reported as a tumor suppressor in lung cancer and can be an independent prognostic marker in LUAD (Zhang et al., 2018; Chen et al., 2021). Furthermore, we showed that CBFA2T3 expression was positively correlated with higher antigen-presenting cell infiltration. Hence, CBFA2T3 can potentially be used as a tumor antigen in future mRNA vaccines. In recent years, tumor antigen receptor chimeric T-cell immunotherapy (CAR-T) has also become a hot topic, and its main mechanism is to engineer T cells isolated from patients to directly recognize and kill tumor cells (Feins et al., 2019). However, due to the low expression of the two genes in lung adenocarcinoma, the benefit of CART-T therapy may not be as good as that of mRNA vaccine, and mRNA vaccine also has the advantages of easier preparation and more economy.

Compared with live attenuated and inactivated vaccines, mRNA vaccines reduce problems associated with endotoxin and infection, and mRNA vaccines do not cause insertional mutagenesis caused by genomic integration compared with DNA vaccines and viral vector-based vaccines (Kim et al., 2021). Although mRNA vaccines are promising, they are easily degraded by extracellular ribonucleases (RNases) due to the difficulty of mRNA as an anionic molecule to counteract the electrostatic repulsion of cell membranes (Kowalski et al., 2019). Therefore, the design of delivery vectors is the key to whether mRNA vaccines can work.

Identification of different immune subtypes contributes to the development of precise treatment strategies (Charoentong et al., 2017). Thus, we classified LUAD patients by immune-related gene expression and identified four immune subtypes with different prognoses. Also, we described the different immune subtype landscapes and analyzed the potential benefits of using mRNA vaccines for each subtype. Notably, we identified a population of patients suitable for mRNA vaccination. This population was formed by early-stage patients with high immune cell infiltration, high immune checkpoints, immunogenic cell death modulators expression, and low TMB. The most relevant characteristic gene modules of this population were found by WGCNA, and four biomarkers that could be used as vaccination responses were found in these modules. Although previous studies have described LUAD immune subtypes (Wu et al., 2021; Zhao et al., 2021; Zhou et al., 2021), in the present study, we associated immune subtypes and mRNA vaccination for the first time. Drug sensitivity analysis of different immune subtypes showed significant differences in drug sensitivity among different subtypes, not only providing a reference for treatment strategies for populations not suitable for vaccination but also revealing the importance of individualized treatment.

Overall, we provided new ideas for the development of mRNA vaccines for LUAD treatment. Further, detailed analyses of immune subtypes revealed the characteristics of the population suitable for mRNA vaccination and emphasized the importance of personalized treatment. Although there have been previous tumor antigen-related studies of other cancers (Huang et al., 2021a; Huang et al., 2021b; Ye et al., 2021b; Zhong et al., 2021), the study of lung adenocarcinoma is the first, our study also reveals the characteristics of the appropriate vaccinated population and finds optional prognostic biomarkers for the potential vaccinated population. In addition, we provide a reference for potentially beneficial treatment strategies through drug sensitivity analysis of lung adenocarcinoma patients with different immune subtypes and finally emphasize the importance of individualized treatment.

## Data Availability

The original contributions presented in the study are included in the article/Supplementary Material, further inquiries can be directed to the corresponding author.

## Abbreviations

LUAD, lung adenocarcinoma; TCGA, The Cancer Genome Atlas; GSVA, gene set variation analysis; ssGSEA, single-sample gene set enrichment analysis; APCs, Antigen-presenting cells; IC50, Half maximal inhibitory concentration; GO, Gene oncology; KEGG, Kyoto Encyclopedia of Genes and Genomes; ICP, Immune checkpoint; ICD, Immunogenic cell death modulators.
