# Supplementary figures and images for: Identification of Tumor Antigens and Immune Subtypes in Lung Adenocarcinoma for mRNA Vaccine Development
Source: Front Cell Dev Biol. 2022 Feb 21;10:815596. doi: 10.3389/fcell.2022.815596 (PMC8899518; doi:10.3389/fcell.2022.815596)

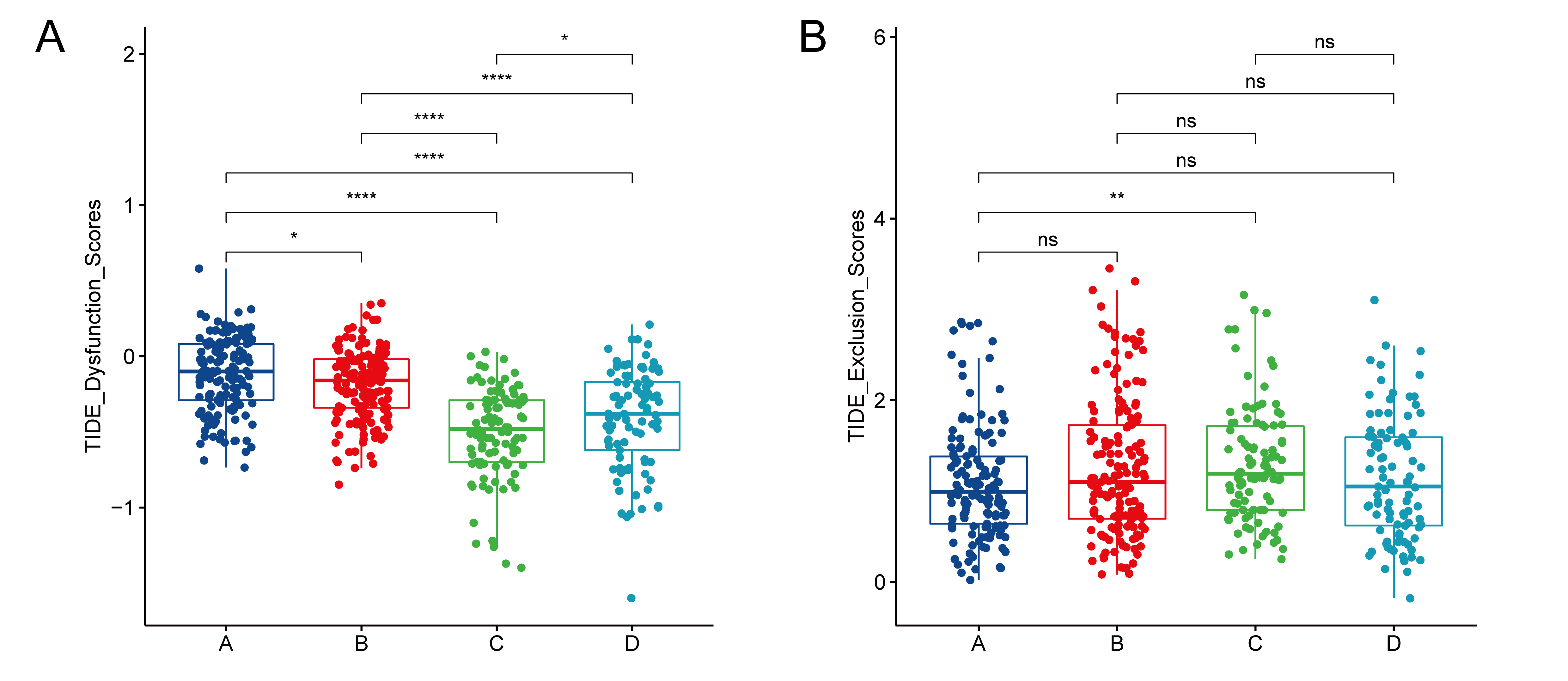

Supplement: Supplementary file 1 [file Image1.TIF]
